# *Pathogens*: Journal Section Readjustment

**DOI:** 10.3390/pathogens10010072

**Published:** 2021-01-15

**Authors:** Lawrence S. Young

**Affiliations:** Warwick Medical School, University of Warwick, Coventry CV4 7AJ, UK; L.S.Young@warwick.ac.uk

The impact of infectious diseases on human life is incalculable. This has been reinforced by the current COVID-19 pandemic, with almost 90 million cases of SARS-CoV-2 coronavirus infection worldwide so far and nearly 2 million deaths. The on-going effect of this pandemic on human health globally and on the world economy has emphasized the importance of research into the biology, epidemiology and clinical management of infectious disease, as well as the development of vaccines and other interventions. Our current situation has also emphasized the need to better understand infectious disease in animals and how *pathogens* spread into the human population. To address this growing interest and focus on *pathogens*, and to further develop the *Pathogens* journal, we have reorganized the journal’s sections based on the different types of infectious agents and on immunological, therapeutic and preventative aspects. The journal will now be divided into the following sections:

- Bacterial *Pathogens*;
- Fungal *Pathogens*;
- Viral *Pathogens*;
- Parasitic *Pathogens*;
- Emerging *Pathogens*;
- Prions;
- Ticks;
- Immunological Responses and Immune Defense Mechanisms;
- Vaccines and Therapeutic Developments.

We hope that our readers and authors will find the new sections helpful and that this restructuring will better represent the diversity and broad appeal of the journal. 

